# A two-step approach combining the Gompertz growth model with genomic selection for longitudinal data

**DOI:** 10.1186/1753-6561-4-s1-s4

**Published:** 2010-03-31

**Authors:** Ricardo Pong-Wong, Georgia Hadjipavlou

**Affiliations:** 1 The Roslin Institute and R(D)SVS, University of Edinburgh, Roslin, Midlothian, EH25 9PS, UK

## Abstract

**Background:**

We used the Gompertz growth curve to model a simulated longitudinal dataset provided by the QTLMAS2009 workshop and applied genomic evaluation to the derived model parameters and to a model-predicted trait value.

**Results:**

Prediction of phenotypic information from the Gompertz curve allowed us to obtain genomic breeding value estimates for a time point with no phenotypic records. Despite that the true model used to simulate the data was the logistic growth model, the Gompertz model provided a good fit of the data. Genomic breeding values calculated from predicted phenotypes were highly correlated with the breeding values obtained by directly using the respective observed phenotypes. The accuracies between the true and estimated breeding value at time 600 were above 0.93, even though t600 was outside the time range used when fitting the data. The analysis of the parameters of the Gompertz curve successfully discriminated regions with QTL affecting the asymptotic final value, but it was less successful in finding QTL affecting the other parameters of the logistic growth curve. In this study we estimated the proportion of SNPs affecting a given trait, in contrast with previously reported implementations of genomic selection in which this parameter was assumed to be known without error.

**Conclusions:**

The two-step approach used to combine curve fitting and genomic selection on longitudinal data provided a simple way for combining these two complex tasks without any detrimental effect on breeding value estimation.

## Background

A longitudinal trait is a composite of phenotypes recorded over time which have a complex genetic correlation structure. Different types of non-linear functions have been used to model a time-dependent trait and dissect its genetic components. For instance, the Gompertz model has been used for analysing the polygenic components [1] and growth QTL [2] for live weight in sheep. Genomic selection (GS) commonly refers to a new class of methods for genetic evaluation using very dense marker maps covering the entire genome [3]. The overall trend so far has been that GS increases the accuracy of the breeding values, especially for those individuals without phenotypic information.

The objective of this study was to estimate genomic breeding values for the trait at time 600 (t600), which resided outside the range of longitudinal yield data provided by the QTLMAS2009 workshop. We implemented a two-step procedure in which first the Gompertz function was fitted to the data for each individual and, then genomic selection was performed on the predicted phenotype at t600 and on the parameter estimates derived from the fitted Gompertz curve.

## Methods

### Data

The data provided by QTLMAS2009 is fully described in [4]. It consisted of 100 full-sib families, each with 20 offspring. Half of the offspring (training set) have both phenotype information of yield at 5 distinct time points (0, 132, 25, 397, 530) and genotype data on 453 SNP markers across 5 Morgans. The remaining offspring (candidate set) had only genotype information.

### Procedure

To obtain genomic breeding values for t600, we used an approach composed by two independent steps: Firstly, a Gompertz growth curve was used to model the performance records across time, and to estimate the model descriptors (A, B, C) which best fit the phenotypes of each individual. Secondly, genomic evaluation was applied to obtain genomic estimated breeding values (GEBVs) for t600 using two different methods: I) estimating GEBVs for the model parameters (A, B, C; i.e. 3 GEBVs per individual) and using them to estimate the breeding value for t600 from the Gompertz function; II) predicting the phenotypes at t600 from evaluating the Gompertz function with the estimated parameters and later applying genomic selection on the predicted t600 phenotypes.

### Growth model

The Gompertz equation is of the form: y(t) = Ae^{-e[Be(C-t)/A]}^, where y(t) is the yield at time t; A the final yield; B the maximum growth rate and C the age at maximum growth rate. The curve fitting was implemented using nonlinear regression in SAS [5]. The Gompertz function was fitted to each individual separately to estimate individual model parameters A, B, C. Subsequently, the fitted individual equations were used to predict the trait at t600 (or t600 GEBVs if using the parameter GEBVs).

### Genomic evaluation

A Bayes B type of analysis was used as first described by Meuwissen *et al.*[3]. Under a Bayesian framework the model accounts for the fact that not all SNPs affect the trait in question. The model assumed in the method is:

where **y** is the vector of phenotypes; **b** contains the fixed effects and **X** is its incidence matrix; α_*i*_ is the allelic substitution effect for SNP *i*; **g**_*i*_ is the vector of genotypes (1, 2 & 3 for genotypes 00, 10/01 and 11, respectively) for SNP *i*; and **e** the vector of residuals distributed N(0,). The allelic substitution effects *α* for each SNP are assumed to be from a mixture distribution with probability π of having an effect on the trait and with probability (1- π) of not affecting the trait at all. If the SNP is affecting the trait, its allelic substitution is distributed N(0, ).

The implementation of the model was done using Gibbs sampling. The parameters ,  and π were also calculated in the analysis using flat priors. So far, the implementations of Bayes B reported in the literature have not estimated π, but assumed it was known without error.

For each analysis, a MCMC chain was run and the first 10000 cycles were discarded as burn-in period. Following this, 10000 realisations were collected, each separated by 50 cycles between consecutive realisations. The posterior mean was used as the estimate for each parameter of interest.

## Results and discussion

### Growth model parameters

The Gompertz model provided a good fit of the data (see additional files 1 and 2) with the curve fitted for each individual being statistically significant. To further test how well the Gompertz curve fitted the phenotypic data, phenotypic values were predicted at all 5 time points for which observed phenotypic data was available. The Pearson and Spearman correlations between the true and predicted phenotypic values at t530 were above 0.99, with similar high correlations obtained for the other 4 time points. These high correlations remained when comparing the GEBVs calculated for both the true and predicted phenotypes.

### Estimation of GEBVs for the parameters of the Gompertz curve

**Figure 1:**
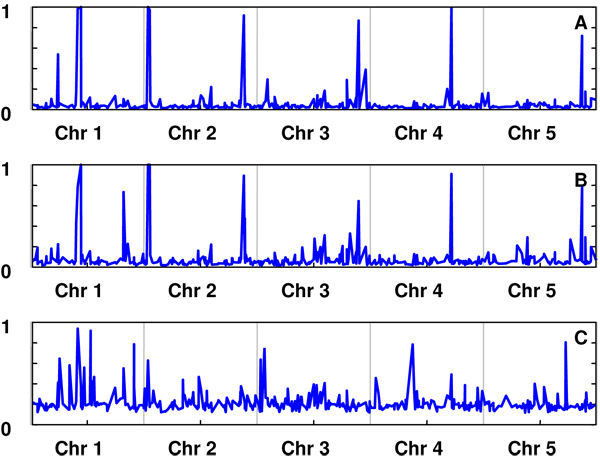
Probability of an individual SNP affecting the parameters A, B or C.

**Figure 2:**
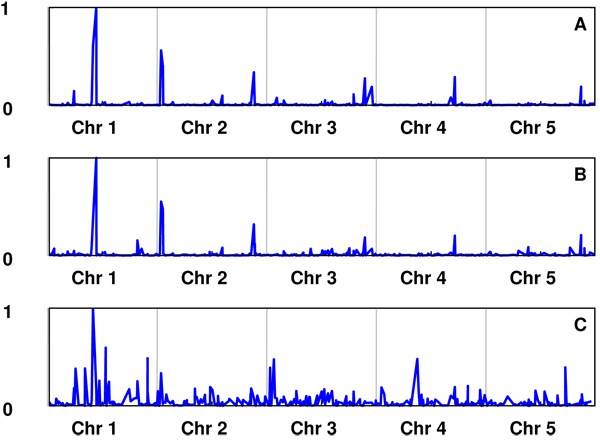
Estimated allele substitution effect for A, B and C. The size of the effects are rescaled relative to the largest allele effect within each parameter (i.e. highest effect=1).

Univariate analyses were performed to each of the three parameters of the Gompertz function. The correlations between the univariate GEBVs for the three parameters were high (correlations between GEBVs for A-B, A-C and B-C were 0.97, 0.71 and 0.59, respectively. The posterior means of π for A, B and C were 0.059, 0.082 and 0.219, respectively. The posterior probabilities for the SNPs having an effect on the parameters A, B and C, and their estimated allelic substitution effects are shown in Figures 1 and 2, respectively. The results suggest that parameters A and B are affected by the same SNPs, with some others affecting parameter C. This is consistent with the high correlation between GEBV for A and B

### Estimation of GEBVs for the trait at a given time point

**Figure 3:**
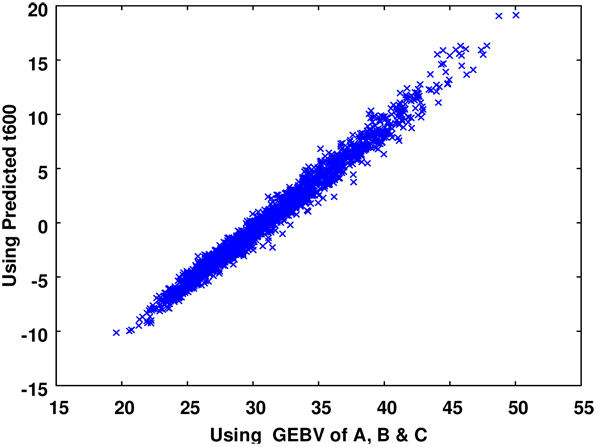
Scatter plot of t600 GEBVs calculated from evaluating the Gompertz function using GEBVs of A, B, C (x-axis) and calculated from Genomic selection on the predicted phenotype at t600 (y-axis). The GEBV not scaled on the same mean.

The GEBVs for t600 obtained by evaluating the Gompertz function with GEBVs for A, B and C (method I) were very similar to those calculated from method II which evaluated the predicted performance at t600 (see Figure 3). The correlation between both approaches for calculating GEBVs was 0.99, with GEBVs from method I having slightly larger variance. The GEBVs obtained from the genomic selection on the predicted trait at t600 show a very similar trend as found for parameters A and B, with the same SNPs of large effect found for A and B also affecting t600 (see additional file 3). The estimate of π for t600 was 0.048.

### Comparison with the true model used to simulate the data

The true model used to simulate the data was the logistic growth curve (see [4]) described as . Both the logistic and the Gompertz models are characterised by an ‘S’ shape growth with an asymptotic maximum yield, but the parameters describing them have different meaning. Comparing both functions, parameters A and *Φ*_1_ have similar definition. The equivalence between the other parameters is less clear, which explains the results obtained. GEBVs for A, B were highly correlated to the true values for *Φ*_1_, but poorly with *Φ*_2_ and *Φ*_3_ (Table 1. For correlations within training and candidate sets see additional file 4). Moreover, the analysis of parameters A and B showed that the SNPs with the highest probability of having an effect on the trait were located at the positions where the six QTL affecting *Φ*_1_were simulated. Locations containing QTL for parameters *Φ*_2_ and *Φ*_3_ were less associated with QTL affecting the parameters of the Gompertz curve.

**Table 1:** Pearson (lower diagonal) and Spearman (upper diagonal) correlations between true and estimated breeding values for t600 and the parameters used to simulate or analyse the data.^1,2^

		**TBV**					**GEBV**		
	**T600**	**Φ_1_**	**Φ_2_**	**Φ_3_**	**t600_I**	**t600_II**	**A**	**B**	**C**
**TBV t600**		0.995	0.230	0.091	0.935	0.937	0.913	0.930	0.405
**TBV Φ_1_**	0.997		0.285	0.160	0.931	0.937	0.928	0.925	0.465
**TBV Φ_2_**	0.291	0.344		0.129	0.237	0.258	0.377	0.306	0.719
**TBV Φ_3_**	0.098	0.157	0.108		0.082	0.112	0.213	0.029	0.463
**GEBV t600_I**	0.942	0.941	0.316	0.079		0.990	0.968	0.979	0.402
**GEBV t600_II**	0.947	0.949	0.332	0.116	0.990		0.969	0.981	0.437
**GEBV A**	0.919	0.933	0.459	0.194	0.970	0.969		0.957	0.599
**GEBV B**	0.938	0.940	0.396	0.034	0.983	0.983	0.971		0.454
**GEBV C**	0.519	0.571	0.735	0.433	0.523	0.551	0.709	0.587	
									

^1^**TBV t600, Φ_1_, Φ_2_ and Φ_2_**: true breeding values for t600, and parameters **Φ_1_, Φ_2_, Φ_2_** from the logistic growth curve. **GEBV t600_I, t600_II, A, B and C:** genomic breeding values for t600 estimated with method I and II and for parameters **A, B** and **C** from the Gompertz curve. Correlations are for all animals.

^2^The results shown here are higher than those presented to the QTLMAS workshop since an error in the implementation was found afterwards. Values presented at the workshop were 0.907 and 0.911 for the Pearson correlations of method I and II, and 0.886 and 0.891 for the Spearman correlations for methods I and II.

Despite that the Gompertz curve was not the true model, its use provided very accurate GEBVs for t600. The correlation between the true breeding values and GEBVs are presented in Table 1 and additional file 4. Both methods of estimating GEBVs yielded similar accuracy. The Pearson and Spearman correlations between true and estimated breeding value with methods I and II for all individuals in the pedigree were above 0.93.

In this study, the proportion of SNPs affecting the trait, π, was estimated in the analysis. This contrasts with previously reported implementations of Bayes B where π was assumed to be known without error. The π values were slightly overestimated, partly due to the low linkage disequilibrium between SNPs (average r^2^ between consecutive SNP was 0.15) and also to the fact that the Gompertz function was not the true model. However, the success in estimating such an important parameter from the data itself, even when assuming a uniform prior, provides an improvement in genomic evaluation relative to assuming that π is known without error.

## Conclusions

The two-step approach of growth model fitting and genomic selection on model parameters and on predicted phenotype appeared to be a simple and reliable strategy. Despite that the Gompertz curve was not the true model used to simulate the data, the correlations between true and estimated breeding values at t600 were very high (Pearson and Spearman correlations above 0.93). The approach of estimating GEBVs for phenotype at a time of interest using GEBVs of the three parameters and evaluating the Gompertz function could be beneficial when GEBVs are needed for different time points. In this study, the proportion of SNP affecting the trait was estimated from the data, contrasting with previous implementation of genomic selection where this proportion has been assumed to be known without error. The results from this study showed that separate implementation of the growth modelling process and genomic evaluation provided huge simplification of the methodology with no detrimental effect on the final results.

## List of abbreviations used

QTL: Quantitative Trait Locus; SNP: Single Nucleotide Polymorphism; GS: Genomic Selection; GEBV: Genomic Estimated Breeding value; MCMC: Monte Carlo Markov Chain

## Competing interests

The authors declare that they have no competing interests.

## Authors' contributions

RPW and GH carried out the analyses and drafted the manuscript. Both authors have read and contributed to the final text of the manuscript.

## Acknowledgements

GH acknowledges the GENACT Project, funded by the Marie Curie Host Fellowships for Early Stage Research Training, as part of the 6^th^ Framework Programme of the European Commission. This work has made use of the resources provided by the Edinburgh Compute and Data Facility (ECDF) (http://www.ecdf.ed.ac.uk/). The ECDF is partially supported by the eDIKT initiative. (http://www.edikt.org.uk).

This article has been published as part of BMC Proceedings Volume 4 Supplement 1, 2009: Proceedings of 13th European workshop on QTL mapping and marker assisted selection.

The full contents of the supplement are available online at http://www.biomedcentral.com/1753-6561/4?issue=S1.

## Supplementary Material

Additional file 1

Additional file 2

Additional file 3

Additional file 4

## References

[B1] Lambe NR Navajas EA Simm G Bunger L A genetic investigation of various growth models to describe growth of lambs of two contrasting breeds. J Anim Sci 2006 84 2642 2654 10.2527/jas.2006-041 16971565

[B2] Hadjipavlou G Bishop SC Age-dependent quantitative trait loci affecting growth traits in Scottish Blackface sheep. Anim Genet 2009 40 165 175 10.1111/j.1365-2052.2008.01814.x 19076734

[B3] Meuwissen THE Hayes BJ Goddard ME Prediction of total genetic value using genome-wide dense marker maps. Genetics 2001 157 1819 1829 11290733 10.1093/genetics/157.4.1819 PMC1461589

[B4] Coster A Bastiaansen J Calus M Maliepaard C Bink M QTLMAS 2009: Simulated Dataset. BMC Proc 2010 4 Suppl 1 S3 10.1186/1753-6561-4-S1-S3 20380757 PMC2857845

[B5] SAS release 9.1 SAS Institute, Cary, NC Ref Type: Computer Program

