# Supplementary figures and images for: A two-step approach combining the Gompertz growth model with genomic selection for longitudinal data
Source: BMC Proc. 2010 Mar 31;4(Suppl 1):S4. doi: 10.1186/1753-6561-4-s1-s4 (PMC2857846; doi:10.1186/1753-6561-4-s1-s4)

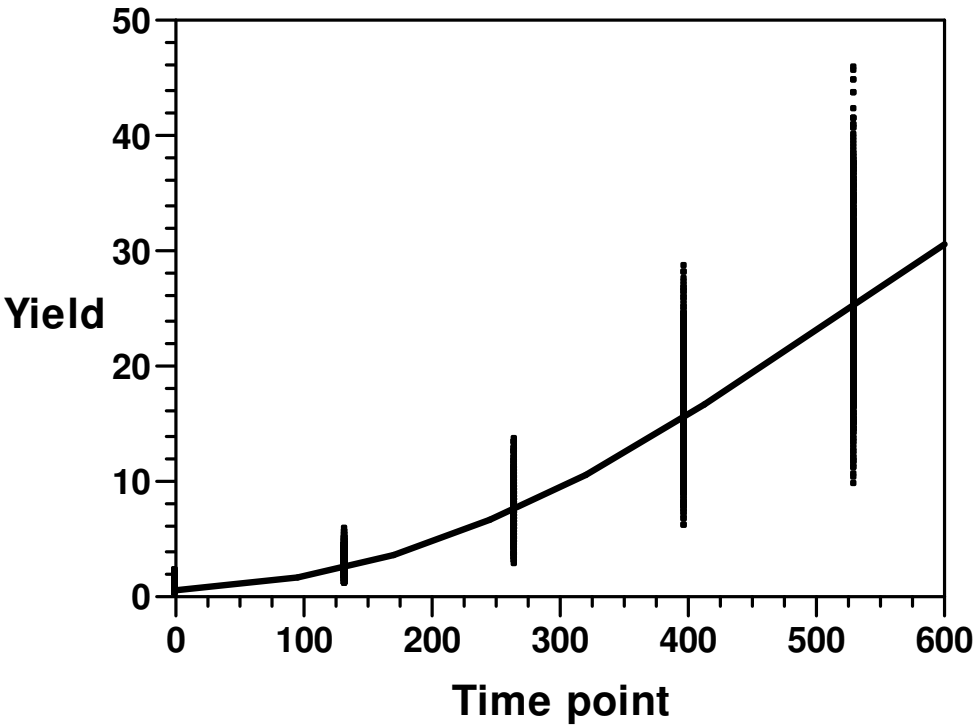

Supplement: Additional file 1 [file 1753-6561-4-S1-S4-S1.pdf]

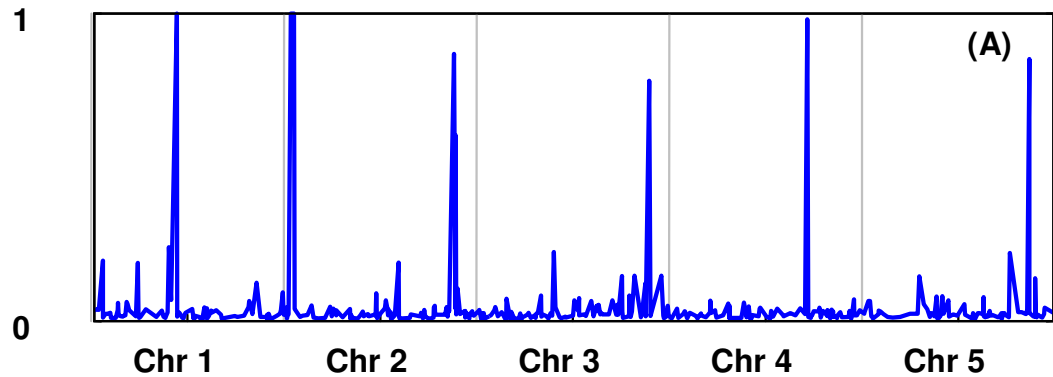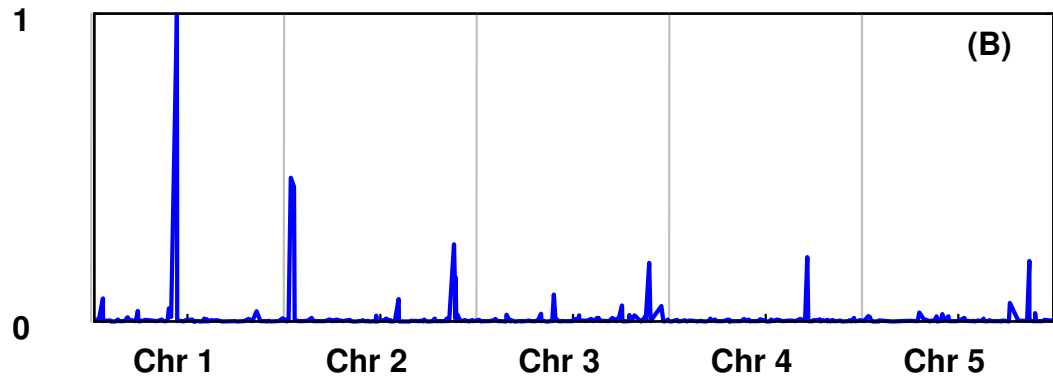

Supplement: Additional file 3 [file 1753-6561-4-S1-S4-S3.pdf]
