# Supplementary material for: A two-step approach combining the Gompertz growth model with genomic selection for longitudinal data
Source: BMC Proc. 2010 Mar 31;4(Suppl 1):S4. doi: 10.1186/1753-6561-4-s1-s4 (PMC2857846; doi:10.1186/1753-6561-4-s1-s4)
Supplement: Additional file 2 [file 1753-6561-4-S1-S4-S2.pdf]

Parameter[1]	Sample mean	Approx. SEM[2]	Approx. 95% Confidence Interval
A (yield)	67.9	0.65	66.58-69.13
B (yield/time unit)	0.076	0.001	0.075-0.077
C (time)	520	1.23	517.8-522.6
Yield at time 600	30.6	0.24	30.11-31.05

[1]The average of individual means, predicted after fitting the Gompertz model to trait values for each individual, was estimated for each parameter.

[2]SEM=Standard Error of the Mean.
