# Supplementary material for: A two-step approach combining the Gompertz growth model with genomic selection for longitudinal data
Source: BMC Proc. 2010 Mar 31;4(Suppl 1):S4. doi: 10.1186/1753-6561-4-s1-s4 (PMC2857846; doi:10.1186/1753-6561-4-s1-s4)
Supplement: Additional file 4 [file 1753-6561-4-S1-S4-S4.pdf]

Unphenotyped offspring (candidate set)[1,2]									
	**TBV**				**GEBV**				
	**t600**	**Φ_1**	**Φ_2**	**Φ_3**	**t600_I**	**t600_II**	**A**	**B**	**C**
**TBV t600**		0.995	0.203	0.036	0.930	0.934	0.899	0.923	0.364
**TBV Φ_1**	0.997		0.259	0.101	0.927	0.934	0.914	0.919	0.422
**TBV Φ_2**	0.292	0.349		0.118	0.235	0.246	0.376	0.284	0.737
**TBV Φ_3**	0.051	0.106	0.093		0.031	0.057	0.153	-0.031	0.389
**GEBV t600_I**	0.940	0.941	0.339	0.040		0.989	0.967	0.979	0.387
**GEBV t600_II**	0.945	0.948	0.350	0.067	0.990		0.965	0.980	0.415
**GEBV A**	0.912	0.928	0.485	0.136	0.971	0.967		0.952	0.584
**GEBV B**	0.935	0.938	0.406	-0.014	0.984	0.984	0.970		0.424
**GEBV C**	0.503	0.555	0.758	0.353	0.533	0.551	0.713	0.586	
**Phenotyped offspring (training set)[1,2]**									
	**TBV**				**GEBV**				
	**t600**	**Φ_1**	**Φ_2**	**Φ_3**	**t600_I**	**t600_II**	**A**	**B**	**C**
**TBV t600**		0.995	0.258	0.139	0.940	0.940	0.926	0.935	0.443
**TBV Φ_1**	0.997		0.311	0.210	0.935	0.939	0.939	0.931	0.503
**TBV Φ_2**	0.294	0.343		0.138	0.243	0.274	0.378	0.330	0.696
**TBV Φ_3**	0.137	0.198	0.132		0.127	0.162	0.268	0.083	0.530
**GEBV t600_I**	0.944	0.941	0.294	0.113		0.991	0.968	0.979	0.417
**GEBV t600_II**	0.949	0.950	0.317	0.157	0.990		0.972	0.981	0.460
**GEBV A**	0.925	0.939	0.435	0.244	0.969	0.970		0.962	0.614
**GEBV B**	0.941	0.941	0.389	0.076	0.983	0.983	0.972		0.482
**GEBV C**	0.534	0.586	0.717	0.500	0.517	0.554	0.707	0.591	
**Parents[1,2]**									
	**TBV**				**GEBV**				
	**t600**	**Φ_1**	**Φ_2**	**Φ_3**	**t600_I**	**t600_II**	**A**	**B**	**C**
**TBV t600**		0.985	0.099	0.092	0.916	0.910	0.885	0.914	0.315
**TBV Φ_1**	0.997		0.179	0.207	0.908	0.906	0.918	0.903	0.424
**TBV Φ_2**	0.182	0.228		0.053	0.063	0.092	0.246	0.130	0.727
**TBV Φ_3**	0.096	0.156	-0.055		0.100	0.124	0.242	0.022	0.443
**GEBV t600_I**	0.960	0.956	0.201	0.047		0.989	0.955	0.965	0.278
**GEBV t600_II**	0.965	0.965	0.201	0.107	0.995		0.965	0.976	0.332
**GEBV A**	0.941	0.956	0.376	0.163	0.963	0.971		0.936	0.502
**GEBV B**	0.960	0.959	0.311	-0.015	0.984	0.983	0.974		0.330
**GEBV C**	0.468	0.527	0.749	0.382	0.411	0.454	0.636	0.502	

[1]**TBV t600, Φ_1, Φ_2 and Φ_2:** true breeding values for t600, and parameters **Φ_1, Φ_2, Φ_2** from the logistic growth curve. **GEBV t600_I, t600_II, A, B and C**: genomic breeding values for t600 estimated with method I and II and for parameters **A, B** and **C** from the Gompertz curve.

[2]The results shown here are higher than those presented to the QTLMAS workshop since an error in the implementation was found afterwards. For the values presented at the workshop, the Pearson correlations between the true and the estimated breeding values of unphenotyped offspring using method I and II were 0.897 and 0.901, and their Spearman correlations were 0.865 and 0.871, respectively. For phenotyped offspring, the Pearson correlations were 0.916 and 0.919 and the Spearman correlations were 0.903 and 0.906, respectively.
